# New Chemical Dopant and Counterion Mechanism for Organic Electrochemical Transistors and Organic Mixed Ionic–Electronic Conductors

**DOI:** 10.1002/advs.202207694

**Published:** 2023-07-19

**Authors:** Vianna N. Le, Joel H. Bombile, Gehan S. Rupasinghe, Kyle N. Baustert, Ruipeng Li, Iuliana P. Maria, Maryam Shahi, Paula Alarcon Espejo, Iain McCulloch, Kenneth R. Graham, Chad Risko, Alexandra F. Paterson

**Affiliations:** ^1^ Department of Chemical and Materials Engineering Department of Electrical Engineering Centre for Applied Energy Research University of Kentucky Lexington KY 40506 USA; ^2^ Department of Chemistry and Centre for Applied Energy Research University of Kentucky Lexington KY 40506 USA; ^3^ Department of Chemistry University of Kentucky Lexington KY 40506 USA; ^4^ Brookhaven National Lab Upton NY 11973 USA; ^5^ Department of Chemistry Chemistry Research Laboratory University of Oxford Oxford OX1 3TA UK; ^6^ King Abdullah University of Science and Technology KAUST Solar Centre Thuwal 23955‐6900 Saudi Arabia

**Keywords:** chemical doping, electron transporting, morphology additive, organic bioelectronics, organic electrochemical transistors, organic electronics, organic mixed ionic–electronic conductors

## Abstract

Organic mixed ionic–electronic conductors (OMIECs) have varied performance requirements across a diverse application space. Chemically doping the OMIEC can be a simple, low‐cost approach for adapting performance metrics. However, complex challenges, such as identifying new dopant materials and elucidating design rules, inhibit its realization. Here, these challenges are approached by introducing a new n‐dopant, tetrabutylammonium hydroxide (TBA‐OH), and identifying a new design consideration underpinning its success. TBA‐OH behaves as both a chemical n‐dopant and morphology additive in donor acceptor co‐polymer naphthodithiophene diimide‐based polymer, which serves as an electron transporting material in organic electrochemical transistors (OECTs). The combined effects enhance OECT transconductance, charge carrier mobility, and volumetric capacitance, representative of the key metrics underpinning all OMIEC applications. Additionally, when the TBA^+^ counterion adopts an “edge‐on” location relative to the polymer backbone, Coulombic interaction between the counterion and polaron is reduced, and polaron delocalization increases. This is the first time such mechanisms are identified in doped‐OECTs and doped‐OMIECs. The work herein therefore takes the first steps toward developing the design guidelines needed to realize chemical doping as a generic strategy for tailoring performance metrics in OECTs and OMIECs.

## Graphical abstract

Organic electrochemical transistor key operating metrics—electron transport, ion transport, and ion–electron coupling—are improved using the Brønsted‐base, tetrabutylammonium hydroxide (TBA‐OH), as a chemical n‐dopant in naphthodithiophene diimide organic mixed conductors. TBA‐OH simultaneously behaves as a dopant and morphology additive to enhance key performance metrics: mobility, volumetric capacitance, and transconductance.

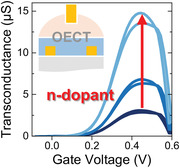

## 1 Introduction

Organic mixed ionic–electronic conductors (OMIECs) are a novel and emergent, yet complex class of organic electronic materials. OMIECs have unique electronic and dynamic structural properties, arising from their ability to couple and simultaneously transport ionic and electronic charges. These intrinsic properties are attracting interest for technologies spanning bioelectronics and medical devices,^[^ ^1^, ^2^ ^]^ neuromorphic applications and computing,^[^ ^3^, ^4^, ^5^, ^6^, ^7^, ^8^, ^9^, ^10^, ^11^ ^]^ as well as energy storage, biological and chemical sensing,^[^ ^12^ ^]^ displays, light emission, and printed circuits.^[^ ^13^ ^]^ This diverse application space is well‐suited to organic electronics: Along with low‐cost, large area, and solution‐processible qualities, a key benefit of organic electronic materials is their range of chemical structures. In stark contrast to inorganic semiconductors and solid conductors, organic electronic materials offer “make‐to‐order” electronics with the electronic and mechanical properties needed to realize the impressively broad OMIEC application space. However, despite this important advantage, to‐date OMIEC research has predominantly focused on a single material, poly(3,4‐ethylenedioxythiophene):polystyrene sulfonate (PEDOT:PSS),^[^ ^14^, ^15^ ^]^ which is infamous for its unalterable structure and poor control of ionic and electronic components.^[^ ^16^ ^]^

To move beyond PEDOT:PSS and fulfill the aforementioned application space, OMIECs with versatile performance metrics are needed. By using the organic electrochemical transistor (OECT) as a tool to benchmark OMIEC parameters,^[^ ^17^ ^]^ chemical synthesis has been shown to be one successful approach for modifying OMIEC performance metrics.^[^ ^18^ ^]^ For example, single‐component, homogenous donor–acceptor copolymers are making excellent progress,^[^ ^19^, ^20^, ^21^ ^]^ including working in operating regimes beyond PEDOT:PSS depletion mode, such as accumulation mode operation for low power applications, and with both electron transporting and ambipolar characteristics for logic circuits.^[^ ^21^, ^22^, ^23^ ^]^ Another approach is to use materials or device engineering strategies.^[^ ^24^ ^]^ For example, the following have been identified for altering OMIEC operation/performance as measured in OECTs: blending materials,^[^ ^25^, ^26^ ^]^ solvent engineering,^[^ ^27^ ^]^ interface modification,^[^ ^28^, ^29^, ^30^ ^]^ and more recently, doping of the bulk active layer during the fabrication steps.^[^ ^31^ ^]^ The latter chemical or molecular doping is of interest for a number of reasons: i) Doping has been critical to the success of silicon electronics and is widely used for traditional organic semiconductors and their devices.^[^ ^32^, ^33^ ^]^ ii) The simplicity of the technique, i.e., admixing a dopant solution with a host polymer solution, is scalable and fits within the low‐cost, solution‐processed framework of organic electronics. iii) Preliminary findings show molecular doping improves OECT transconductance (*g*_m_), charge carrier mobility (*µ*) and volumetric capacitance (C*),^[^ ^31^ ^]^ suggesting chemical doping has the potential to influence key figures of merit underpinning a variety of OMIEC applications.^[^ ^13^ ^]^

If doping has the potential to impact OMIEC‐based electronics in the same way doping revolutionized silicon technologies, how can it be realized as a generic strategy for OECTs and OMIECs? Key reasons that doping is unexploited in OMIECs include a lack of identified dopants that operate in air and water, and lack of design guidelines. Here, we use electron transporting OECTs to approach these challenges. We find the Brønsted‐base, tetrabutylammonium hydroxide (TBA‐OH), successfully operates as both a morphology‐changing additive and a chemical n‐dopant, to enhance key performance metrics in donor–acceptor copolymer naphthodithiophene diimide (pNDTI‐TT) OECTs. In terms of morphology‐changing additive, we find the presence of TBA‐OH may contribute to preserving structural integrity in organic layers that have been exposed to electrolyte. In terms of a chemical n‐doping, the OH^−^ behaves as an anion transfer/exchange n‐dopant and improves *g*_m_, C*, and *µ*. Finally, we identify a preferred “edge‐on” location for the TBA^+^ counterion that reduces its Coulombic interaction with the polaron, to enhance the polaron delocalization. Overall, a new chemical dopant and new design rule are identified for doped‐OECTs and doped‐OMIECs.

## 2 Results

### 2.1 A New Chemical n‐dopant for Organic Electrochemical Transistors and Organic Mixed Ionic‐Electronic Conductors

**Figure 1:**
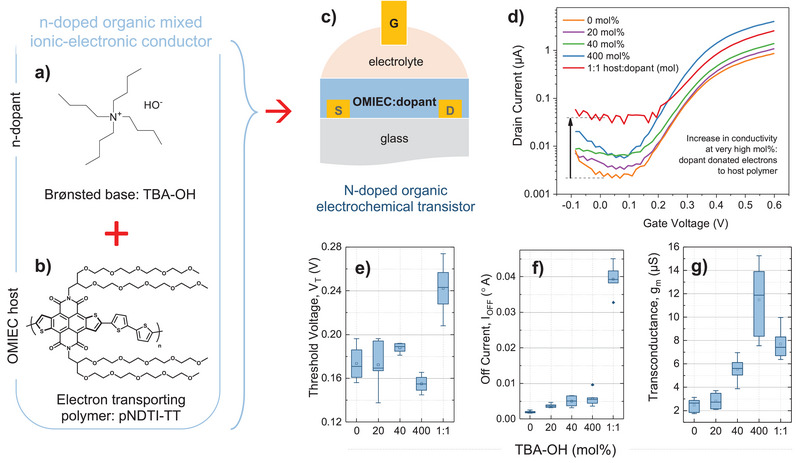
Chemical structure of a) tetrabutylammonium hydroxide (TBA‐OH) and b) naphthodithiophene diimide (pNDTI‐TT), used as the n‐type donor–acceptor copolymer acceptor unit. c) Schematic of a top‐gate bottom‐contact OECT used in this work, indicating that the pNDTI‐TT:TBA‐OH system is used as the organic mixed ionic–electronic conductor active layer. d) Backward sweep transfer characteristics of pNDTI‐TT:TBA‐OH OECTs with 0, 20, 40, 400 mol%, with an additional, highly doped system are included at 1:1. Statistical variation of e) threshold voltage (*V*_T_), f) off‐current (*I*_OFF_), and g) peak transconductance (*g*_m_), with systematically varied quantities of TBA‐OH, taken over six devices each. *V*_T_ has been extracted from the relationship between √*I*_D_ and *V*_G_. The shift in *V*_T_ and gradual improvement in *g*_m_ and *I*_OFF_—that worsen again at very high dopant concentrations—indicate that TBA‐OH successfully n‐dopes the pNDTI‐TT. Although the key performance metrics are improved in the best‐performing 400 mol% system, (e)–(g) show a large deviation in the 400 mol% data set. The latter highlights an important point about device uniformity challenges with disordered, solution‐processed polymers, and more so for OECT active layers transporting ions and swelling when exposed to electrolyte. Figure S3 in the Supporting Information confirms the impact of the dopant at 400 mol%: we fabricated an additional six OECTs, on different days and with pNDTI‐TT synthesized in a different batch, to compare the pristine pNDTI‐TT with the best‐performing pNDTI‐TT:TBA‐OH (400 mol%).

We opted to identify a new chemical n‐dopant, because n‐type OMIECs typically have lower performance than p‐type OMIECs,^[^ ^21^ ^]^ and balanced performance is important for complementary technologies.^[^ ^34^ ^]^ Direct charge transfer n‐dopants have high lying highest occupied molecular orbitals (HOMO), i.e., low ionization energies, to donate electrons to the lowest unoccupied molecular orbital (LUMO) of the host.^[^ ^32^, ^33^, ^34^, ^35^ ^]^ This makes direct charge transfer n‐dopants reactive in oxygen and air and, in general, makes it extremely challenging to identify stable n‐dopants for organic semiconductors. For OECTs, it is more challenging because OECTs should demonstrate operation in aqueous electrolytes, for their intended use in bioelectronic systems. Additionally, OMIEC LUMOs are often shallow (≈−4 eV), to avoid reduction reactions in water.^[^ ^36^ ^]^ One option to bypass this instability is to use a charge‐transfer salt/dopant salt.^[^ ^37^, ^38^, ^39^, ^40^ ^]^ Anion‐induced doping mechanisms have successfully demonstrated stable n‐doping in polymers operating in ambient, aqueous environments; specifically, tetra‐n‐butylammonium fluoride (TBAF) was found to behave as an n‐dopant in naphthalene diimide (NDI)‐based OMIECs.^[^ ^31^ ^]^ We therefore identified a material that was likely to undergo similar anion mechanisms. We selected the OH‐anion in the Brønsted‐base TBA‐OH (**Figure** **1**a), because Kim et al. showed that it will undergo/initiate anion‐induced n‐doping mechanisms in NDI‐based organic thin‐film transistors (OTFTs).^[^ ^40^ ^]^ TBA‐OH is also an attractive option because it is low‐cost and commercially available. Next, we chose a compatible host for the TBA‐OH. The host organic layer is an important consideration for doped‐organic semiconductors (OSCs) because the diverse chemical structures have made doping harder to realize generally, compared to inorganics, in addition to poor organic doping efficiencies, doping technique variation, and morphology changes.^[^ ^41^, ^42^, ^43^, ^44^ ^]^ Based on the aforementioned success of TBA‐OH in NDI‐based OTFTs,^[^ ^40^ ^]^ we chose a recently developed, n‐type donor–acceptor copolymer, with a thiophene‐annulated derivative of NDI as the acceptor unit, naphthodithiophene diimide (pNDTI‐TT) (Figure 1b),^[^ ^45^ ^]^ as the host OMIEC. We note that both materials are highly soluble, and therefore suited to the simple, cost‐effective solution‐doping technique that will be used in this work.^[^ ^40^ ^]^

OECTs were then used to explore the systematic impact of TBA‐OH in pNDTI‐TT at different doping concentrations (Figure 1c–g). The importance of this experiment is several fold: i) Transistors are exceptional tools for exploring fundamental effects of doping on charge transport, as well as identifying new dopant materials;^[^ ^43^ ^]^ ii) OECTs are used to benchmark OMIEC performance metrics,^[^ ^17^ ^]^ iii) OECTs are popular, practical devices, identified as fundamental building blocks for advanced electronics, body–machine interfaces, drug delivery, smart textiles, bioelectronics, adaptive healthcare, and neuromorphic computing;^[^ ^16^, ^46^, ^47^, ^48^, ^49^ ^]^ iv) doping benefits transistors, as is evident from traditional field‐effect transistors, by filling traps, enabling threshold voltage control, minimizing contact resistance, increasing measured mobility, and improving bias stress stability.^[^ ^33^, ^41^, ^44^ ^]^ Two solutions containing TBA‐OH and pNDTI‐TT, dissolved in methanol (as purchased) and chloroform, respectively, were admixed, at varying concentrations of TBA‐OH to the total molar mass of the pNDTI‐TT: 0, 20, 40, 400 molar percentage (mol%). A further highly doped solution at 1:1 solution volume ratio was included to confirm/explore doping trends on transistor performance.^[^ ^50^ ^]^ The TBA‐OH concentrations were chosen to give a broad overview of the impact TBA‐OH has on OECT device performance, as well as to identify the optimum dopant concentration. To normalize any possible aggregation effects arising from the minor solvent blend, exactly the same quantities of methanol and chloroform were used in each solution, regardless of the TBA‐OH concentration.^[^ ^41^, ^42^, ^50^, ^51^, ^52^, ^53^, ^54^ ^]^ The solutions were left overnight in a nitrogen‐filled glovebox and spin‐coated into OECT structures with 600 and 30 µm channel lengths and widths, respectively. The film thicknesses were measured in the OECT channels using either atomic force microscopy (AFM) or a Dektak profilometer. 1 m NaCl_(aq.)_ was used as an electrolyte with Ag/AgCl gate electrodes. For each system, at least six OECTs were fabricated and tested, to give statistical trends that account for the natural variation in OECT device uniformity. Figure 1c–g and Figures S1 and S2 in the Supporting Information show the systematic trend TBA‐OH has on pNDTI‐TT OECTs. The trends shown are a textbook representation of doped transistors: The threshold voltage (*V*_T_) shifts toward zero at the best‐performing concentration (400 mol%) and increases again at 1:1; the off‐current (*I*_OFF_) gradually increases and is highly pronounced at 1:1; and the on‐current (*I*_ON_) gradually increases before decreasing again at 1:1. Above the best‐performing 400 mol%, there are likely adverse scattering effects between charge carriers and dopant molecules, as well as disruption to the morphological structure that explains the reduction in performance at 1:1. Importantly, we find that TBA‐OH indeed impacts *g*_m_ (Figure 1g and Figure S1a, Supporting Information), *µ* (Figure S1b, Supporting Information), and C* (Figure S1d,e, Supporting Information).

Although key performance metrics are improved in the best‐performing 400 mol% system, there is a large deviation in the 400 mol% data set (Figure 1e–g). The latter highlights the importance of device uniformity in disordered, solution‐processed polymers, and even more so for OECT active layers transporting ions and swelling in electrolyte. To verify the impact of the TBA‐OH at 400 mol%, we fabricated an additional six OECTs, with a newly synthesized batch of pNDTI‐TT, comparing pristine pNDTI‐TT with the best‐performing pNDTI‐TT:TBA‐OH (400 mol%). Figure S3 in the Supporting Information and **Table** **1** show the addition of TBA‐OH improves OECT *g*_m_ by up to 9×, from 4.8 µS to a maximum 43.1 µS, when compared to the pristine system. Furthermore, *µ* increases from 9.8 ×10^−4^ to 3.4 ×10^−3^, C* increases from 151.3 to 589.8 F cm^−3^, and the µC* product extracted from the OECTs increases by >13×, from 0.15 to 2.0 F cm^−1^ V^−1^ s^−1^. The increase in *µ* may occur because the TBA‐OH donates additional charge carriers to the pNDTI‐TT. On the one hand, these additional carriers act to fill trap states (evidence for this is that *V*_T_ shifts with the addition of dopant). On the other hand, additional carriers impact bulk charges on the polymer film, which in turn draws in additional ions.^[^ ^31^ ^]^ C* may be improved because the TBA‐OH increases charge carrier density and hence capacitance; additionally, we see a gradual reduction in the thin‐film thickness with increasing TBA‐OH concentrations,^[^ ^42^, ^54^ ^]^ from 65.5 nm in the pristine system to 30.1 nm in pNDTI‐TT:TBA‐OH (400 mol%) (Table S1, Supporting Information). C* is therefore likely increased because of these combined effects. **Table** **2** compares normalized *g*_m_ for the best‐performing pNDTI‐TT:TBA‐OH (400 mol%) to other n‐type systems in the literature. We find that the best‐performing pNDTI‐TT:TBA‐OH (400 mol%) OECTs have the third highest transconductance for all n‐type OECTs reported to‐date. Overall, this highlights the remarkable effectiveness of the TBA‐OH for significantly enhancing key performance metrics,^[^ ^13^ ^]^ via a simple processing technique.

**Table 1:** pNDTI‐TT:TBA‐OH OECT performance metrics. The performance metrics in the table are averaged over six devices. The mobility value is taken from the transconductance

System		Performance metrics						
Polymer	TBA‐OH [mol%]	*g*_m_ [µS]	*V*_T_ [V]	C* [F cm^−3^]	*µ* [cm^2^ V^−1^ s^−1^]	µC*	*I*_ON_ [A]	*I*_OFF_ [A]
NDTI	0	4.79 ± 1.1	0.22	151.33	9.83 × 10^−4^	0.149	8.29 × 10^−7^	1.13 × 10^−9^
	400	43.1 ± 2.1	0.21	589.76	3.36 × 10^−3^	1.983	1.13 × 10^−5^	1.38 × 10^−8^

**Table 2:** Figures of merit—the transconductance (*g*_m_) and the µC* product—compared to published values for other n‐type OECTs in the literature. Each *g*_m_ is normalized (S cm^−1^) for the three channel dimensions, channel width (*W*), length (*L*), and thickness (*d*), where the values used are taken from the literature and reported in the table. The µC* products are extracted from *g*_m_, except in ref. [55] where the µC* product is calculated with the saturation *µ* extracted from √*I*_D_ versus *V*_G_. Biasing conditions used to report the figures of merit are listed

Material system	*g*_m_ normalized for *W*, *d*, and *L* [S cm^−1^]	µC* [F cm^−1^ V^−1^ s^−1^]	Channel geometry: *L*, *W* [µm]	Film thickness [nm]	Biasing conditions [V]	Reference
NDTI:TBA‐OH (400 mol%)	0.454	1.98	30, 600	47	*V*_D_ = 0.6 V *V*_G_ = 0 to 0.6 V	This work
p(gNDI‐gT2)	0.013	0.06	10, 100	55	*V*_D_ = 0.6 V *V*_G_ = 0 to 0.6 V	[56]
p(C3‐gNDI‐gT2)	0.034	0.13	10, 100	57	*V*_D_ = 0.6 V *V*_G_ = 0 to 0.6 V	[56]
p(C6‐gNDI‐gT2)	0.037	0.16	10, 100	46	*V*_D_ = 0.6 V *V*_G_ = 0 to 0.6 V	[56]
P‐90:TBAF (40 mol%)	0.091	0.03	10, 100	116	*V*_D_ = 0.6 V *V*_G_ = 0 to 0.6 V	[57]
P‐100:TBAF (40 mol%)	0.154	N/A	10, 100	50	*V*_D_ = 0.6 V *V*_G_ = 0 to 0.6 V	[57]
BBL	0.3	0.65	20, 39 000	180	*V*_D_ = 0.7 V *V*_G_ = 0 to 0.7 V	[58]
BBL	0.8	1.99	10, 100	80	*V*_D_ = 0.7 V *V*_G_ = −0.1 to 0.6 V	[59]
PgNaN	0.212	0.65	10, 100	1500	*V*_D_ = 0.4 V *V*_G_ = 0 to 0.4 V	[55]
PgNgN	0.007	0.05	10, 100	500	*V*_D_ = 0.4 V *V*_G_ = 0 to 0.4 V	[55]
PBFDO	1970	180	1000, 1000	56	*V*_D_ = 0.6 V *V*_G_ = −0.5 to 0.6 V	[60]

**Figure 2:**
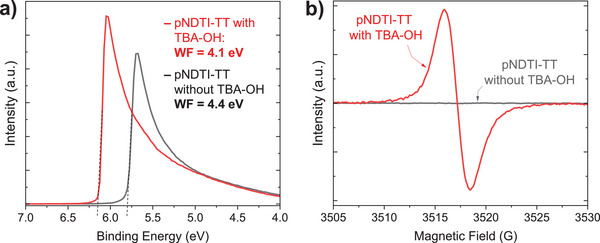
a) UPS showing the secondary electron cut‐off region with and without 400% TBA‐OH (*hν =* 10.2 eV). b) EPR spectra showing a significant increase in signal intensity with the addition of the TBA‐OH to pNDTI‐TT, as compared to pNDTI‐TT without any TBA‐OH.

To confirm that n‐doping is indeed occurring with the addition of TBA‐OH, we used ultraviolet photoelectron spectroscopy (UPS) and electron paramagnetic resonance (EPR) spectroscopy. UPS was used to identify TBA‐OH as an n‐dopant for OTFTs by Kim et al., who measured a Fermi level shift toward the conduction band,^[^ ^40^ ^]^ while EPR has been used to detect Lewis acid doping in semiconductors.^[^ ^31^, ^42^, ^54^, ^61^, ^62^, ^63^, ^64^ ^]^ The UPS results are shown in **Figure** **2**a and Figure S4a in the Supporting Information. The work function decreases from 4.4 eV in the pristine (0 mol%) system to 4.1 eV for the best‐performing (400 mol%) TBA‐OH:NDTI system (Figure 2a), which is a characteristic behavior of n‐type doping.^[^ ^65^ ^]^ Considering that the band gap of pNDTI‐TT is 1.30 eV,^[^ ^45^ ^]^ a 0.3 eV shift in the work function and a 0.1 eV shift in the HOMO onset away from the Fermi energy (Figure S4a, Supporting Information) provide strong evidence that pNDTI‐TT is n‐doped by TBA‐OH. The EPR results provide further evidence that pNDTI‐TT is n‐doped by TBA‐OH. Here, Figure 2b shows a pronounced increase in the EPR signal indicating that TBA‐OH generates unpaired electrons in pNDTI‐TT.^[^ ^42^ ^]^ We attribute this phenomena to the OH^−^ anion, as supported by studies showing how anion/cations can transfer from Lewis acids, Lewis bases, and Brønsted‐bases to behave as p‐ or n‐dopants in various organic semiconductor families.^[^ ^40^, ^66^, ^67^, ^68^, ^69^ ^]^ Indeed, n‐doping can increase the measured *µ* closer to that of the intrinsic material *µ*, and increase the number of charge carriers for charge storage/C*, which in turn improve the µC* product and therefore *g*_m_. In the case of TBA‐OH:NDTI, the OH^−^ anion transfers from the TBA‐OH and produces a hydroxide‐polymer segment, which in turn releases an electron to a hydroxide‐free polymer segment, and by doing so acts as an n‐dopant.^[^ ^31^, ^40^, ^66^, ^67^, ^68^, ^69^ ^]^ We therefore suggest the following reaction mechanism for TBA‐OH and pNDTI‐TT

(1) $$\left[\mathrm{TBA}-\mathrm{OH}\right]\to {\mathrm{TBA}}^{+}+OH^{-}$$

(2) $${\mathrm{OH}}^{-}+P\to {\left[P-\mathrm{OH}\right]}^{-}$$

(3) $${\left[P-\mathrm{OH}\right]}^{-}+P\to \left[P-\mathrm{OH}\right]+P^{-}$$

(4) $$P^{-}+\mathrm{TB}A^{+}\to {[\mathrm{TBA}}^{+}-P^{-}]$$

(5) $$\left[\mathrm{TBA}-\mathrm{OH}\right]+2P\to {[\mathrm{TBA}}^{+}-P^{-}]+[P-\mathrm{OH}]$$

where the chemical equation for electrochemical doping with hydrated cations (C^+^) and charge carriers (e^−^), in the presence of the aforementioned, two‐step chemical doping, is as follows

(6) $$\begin{array}{ccc} \mathrm{pNDTI} & - & TT^{0}\;+\;C^{+}\;+\;{\left[\mathrm{pNDTI}-\mathrm{TT}:\mathrm{OH}\right]}^{-}\\ & & \to \;{\left[\mathrm{pNDTI}-\mathrm{TT}:\mathrm{OH}\right]}^{0}+\;\mathrm{pNDTI}-TT^{-}+C^{+} \end{array}$$

and the pNDTI‐TT‐hydroxide is assumed to be decomposed.^[^ ^70^ ^]^

### 2.2 Chemical Dopants Behave as Morphology Directing Additives

**Figure 3:**
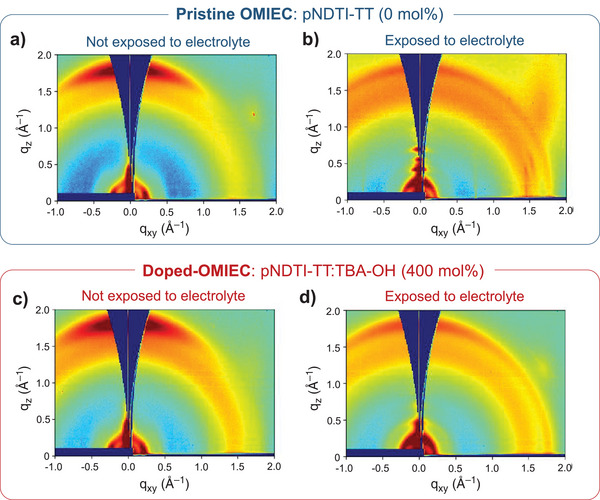
GIWAXS data for the pristine pNDTI‐TT system (i.e., 0 mol% TBA‐OH) thin film, measured when a) not exposed to electrolyte and b) after exposure to 0.1 m NaCl electrolyte for 3 days. GIWAXS data for the best‐performing pNDTI‐TT doped with 400 mol% TBA‐OH measured when c) not exposed to electrolyte and d) after exposing to 0.1 m NaCl electrolyte for 3 days.

Another potentially lucrative, yet complex, feature of doped organic systems is that the dopant may simultaneously behave as a morphology directing additive. Dopant‐directed morphology modifications in other chemically doped organic systems have been found to be beneficial for charge transport.^[^ ^42^, ^54^ ^]^ In the case of OECTs, the performance may also be enhanced if the dopant‐directed morphology modification enables more balanced ionic and electronic charge transport.^[^ ^71^ ^]^ Therefore, to explore the impact of TBA‐OH on morphology in the pNDTI‐TT thin films, we used grazing‐incidence wide‐angle X‐ray scattering (GIWAXS) and AFM, to compare pristine pNDTI‐TT to the best‐performing pNDTI‐TT:TBA‐OH (400 mol%). To account for any subsequent changes in morphology that occur because of swelling, we also compared the pristine and 400 mol% systems after they had been exposed to the 0.1 m NaCl electrolyte. The exposed films may be better representative of the morphology during OECT operation, when compared to the dry films. **Figure** **3** and Figures S5 and S6 in the Supporting Information show the GIWAXS results. We find both pristine and best‐performing doped microstructures are similar before they are exposed to electrolyte, with *π*–*π* stacking of pNDTI‐TT (*d* = 0.35 nm) occurring primarily in the out‐of‐plane direction, and lamellar stacking (*d* = 2.8 nm) of pNDTI‐TT in the in‐plane direction. After the films are exposed to electrolyte, the pristine system has structure features and in‐plane peaks at *d* = 0.82 and 0.41 nm (first and second ordering, respectively) perpendicular to the lamellar backbones (Figure S6c, Supporting Information). The presence of these subtle features suggest there is higher crystallinity in the pristine system after exposure to electrolyte, when compared to the best‐performing pNDTI‐TT:TBA‐OH (400 mol%). In both pristine and pNDTI‐TT:TBA‐OH (400 mol%), the orientation becomes more isotropic after exposure to electrolyte, and the dominant lamellar stacking direction shifts from favoring in‐plane stacking to slightly favoring out‐of‐plane stacking. The d‐spacing of the *π*–*π* stacking remains the same as the system that has not been exposed to electrolyte. After electrolyte exposure, the pristine 0 mol% system out‐of‐plane lamellar spacing increases from 2.6 to 4.6 nm, whereas the pNDTI‐TT:TBA‐OH (400 mol%) maintains a d‐spacing of 2.6 nm—about half the lattice of the pristine system. The pristine system also shows higher crystallinity with more ordering in the lamellar packing, and new in‐plane structural features (*d* = 0.82 and 0.41 nm, in Figure S6c, Supporting Information), whereas the pNDTI‐TT:TBA‐OH (400 mol%) has no in‐plane features. The lack of in‐plane features for the latter has previously been associated with high planarity and no preferential cofacial alignment, which enables strong intermolecular *π*–*π* interactions with tighter d‐spacing.^[^ ^45^ ^]^

The GIWAXS indicates a major backbone orientation change after exposure to electrolyte, in systems with and without TBA‐OH, with the lamellar spacing in the TBA‐OH‐doped polymer half that of the pristine polymer. AFM imaging in Figure S7 (Supporting Information), along with respective root mean square (RMS) data in Figure S8 (Supporting Information), further supports TBA‐OH changing the pNDTI‐TT morphology, and confirming its role as a morphology additive. Figure S8 and Table S1 in the Supporting Information show that the presence of TBA‐OH in pNDTI‐TT results in a decrease in overall film thickness, and increase in surface roughness, indicating that doping has a strong impact on the OMIEC layer and may cause the morphology to be denser and more aggregated. Dopant‐directed morphology modifications may be beneficial because they enable ions to infuse more easily into the polymer film, resulting in efficient/effective electrochemical doping.^[^ ^66^ ^]^ However, we note that disentangling the precise mechanism behind dopants as morphology additives, i.e., convoluted dopant–structure–property relationships, is extremely challenging. The complexity of this topic is well‐documented for organic semiconductors,^[^ ^42^, ^54^, ^73^, ^74^, ^75^ ^]^ and is more challenging in OMIECs, where electrolyte solvent and ions are present during device operation.^[^ ^76^, ^77^ ^]^ Figure S9 in the Supporting Information verifies that the dopant‐directed morphology modifications do not have a significant impact on the pNDTI:TBA‐OH (400 mol%) OECT operational stability. After applying a constant voltage (*V*_G_ = *V*_D_ = 0.4 V) for 2650 s, the pNDTI:TBA‐OH (400 mol%) maintains a high current (≈2.4 µA) as compared to the pristine system (≈0.48 µA). The overall reduction in *I*_D_ is similar for both systems, with *I*_D_ reducing by 7.0% for the pristine polymer, compared to 7.6% in pNDTI:TBA‐OH (400 mol%). We note that this system has excellent potential for further optimization in terms of stability and performance. The 400 mol% identified as the best‐performing system here is dependent on host polymer, processing parameters, and solvent, where solvent choice can impact doping efficiency.^[^ ^42^, ^54^ ^]^ Additionally, the relatively high, best‐performing doping concentration is attributed to the solution‐doping technique used in this work that was chosen for its simplicity.^[^ ^73^, ^78^ ^]^

### 2.3 Identifying a New Counterion Mechanism for Doped Organic Electrochemical Transistors and Doped Organic Mixed Ionic‐Electronic Conductors

**Figure 4:**
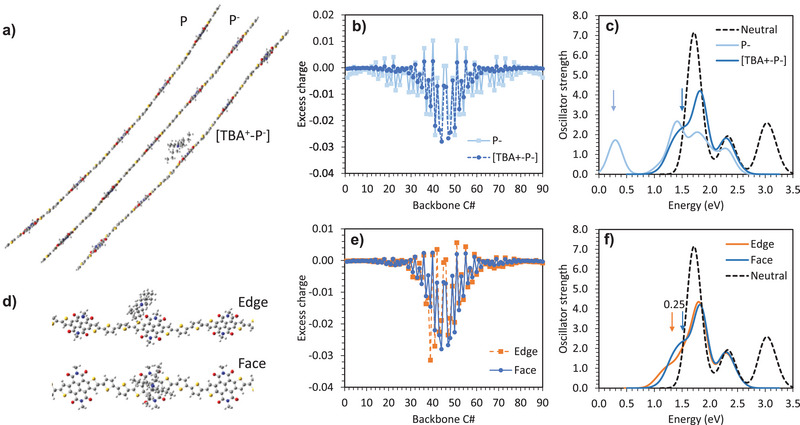
Characteristics of doped pNDTI‐TT oligomer obtained by DFT calculations: a) chain geometry of neutral state P, reduced state without counterion P^−^, and reduced state with counterion [TBA^+^‐P^−^]; b) polaron charge distribution for P^−^ and [TBA^+^‐P^−^]; c) optical absorption spectrum for P, P^−^, and [TBA^+^‐P^−^], the position of the polaron peak shown by the arrow reflects how strongly bound the polaron is; d) “edge” and “face” locations of TBA^+^ counterion relative to pNDTI‐TT oligomer (chains truncated for clarity); the associated polaron charge distributions are shown in (e) and optical absorption spectra in (f). We note that, although there is no substantial, visible difference in the polaron delocalization, the shape of the polaron charge distribution is noticeably different, as influenced by the position of the counterion relative to the pNDTI‐TT unit where the polaron is centered, as shown in (e).

So far, the GIWAXS and AFM data indicate TBA‐OH preferentially alters morphology, while the combined OECT, UPS, and EPR data provides strong evidence that TBA‐OH is an excellent n‐dopant for the pNDTI‐TT OMIEC. However, the question arising from the aforementioned reaction mechanism is: what happens to the TBA^+^ counterion? Although unexplored in doped OECTs or OMIECs, the counterion created after chemical doping in low dielectric constant organic semiconductors is known to impact electronic properties.^[^ ^38^, ^39^, ^79^, ^80^, ^81^, ^82^, ^83^ ^]^ We therefore used density functional theory (DFT) to further investigate the role of the TBA^+^ counterion, with the aim of establishing whether this should be considered as a design guideline/criterion for doped‐OECTs and doped‐OMIECs going forward. **Figure** **4** summarizes the results from the DFT calculations on single oligomers. We find that the polaron charge distribution in pNDTI‐TT is substantially narrower with the TBA^+^ counterion, compared to without, where the full‐widths at half‐maximum are 9 and 12 backbone double bonds, respectively. The polaron localization, in the presence of the counterion, is caused by the Coulomb interaction between the polaron and the counterion, which yields a more stable/bound polaron. This is shown by the large blue shift in the main sub‐gap polaron feature in the optical absorption spectrum, of a reduced chain with and without a counterion presence (Figure 4c), where the polaron peak shifts by ≈1.2 eV when the counterion is present. Indeed, more bound polarons are more localized and their energy levels are more shifted compared to the frontier orbitals.^[^ ^84^ ^]^ Optical transitions involving the polaronic states and the frontier orbitals can be observed by UV‐vis‐IR spectroscopy,^[^ ^85^ ^]^ and the excitation energies associated with these transitions reflect the stability of the polaron. Interestingly, the DFT analysis also shows the position of the TBA^+^ relative to the polymer chain impacts the polaron characteristics: Polarons are less bound if the TBA^+^ is located at the edge of the conjugated backbone (“edge‐on” location), compared to the face of the backbone (“face‐on” location), as supported by the substantial red shift (up to 0.25 eV) in the optical absorption spectrum (Figure 4f). This is rationalized by the longer counterion–polaron distance associated with the edge‐on location, which reduces the Coulomb interaction responsible for stabilizing the polaron. Given that lamellar spacing and *π*‐stacking can keep the counterion further away from the polymer backbone, where the polaron is located, the Coulombic interactions between polarons and counterions are reduced in systems with more pronounced crystalline features.^[^ ^81^, ^86^ ^]^ The latter effect is important for achieving high mobilities.^[^ ^81^, ^82^ ^]^ In this case, the GIWAXS indicates that pNDTI‐TT containing TBA‐OH has closer d‐spacing as compared to pristine pNDTI‐TT. This may result from the TBA^+^ being intercalated between lamella stacks in the preferential edge‐on location, which in turn may reduce the polymer swelling when exposed to electrolyte. Furthermore, TBA^+^ is a large counterion and may preferentially adopt the edge‐on position with its center of mass further away, thereby reducing interactions with polarons. This preferential edge‐on counterion position has not been reported before for chemical or molecular dopants in OECTs or OMIECs.

Finally, we tested the general applicability of TBA‐OH as an n‐dopant by exploring its impact on OECTs in another host polymer. We chose naphthalene diimide (pNDI‐TT) as a host (Figure S10a, Supporting Information), based on its likelihood to undergo the aforementioned reaction mechanism,^[^ ^40^ ^]^ and tested OECTs with TBA‐OH at 0, 40, 400 mol% and 1:1 volume ratio, to compare with pNDTI‐TT. We note that pNDI‐TT has lower intrinsic performance metrics than pristine pNDTI‐TT in OECTs,^[^ ^45^ ^]^ and this experiment is designed to explore systematic changes in OECT or trends, not specific parameter values. Figure S10 in the Supporting Information shows the OECTs exhibit certain characteristic symbols of doped transistors: *V*_T_ shift toward 0 V with the addition of TBA‐OH, *I*_ON/OFF_ improvement, improvement in device uniformity. Additionally, thin‐film color changes at 1:1 (Figure S10b, Supporting Information), indicating adducts/charge transfer complexes are formed between the TBA‐OH (i.e., the OH^−^) and pNDI‐TT.^[^ ^54^, ^62^ ^]^ However, although performance metrics (*g*_m_, C*, and *µ*) are changed, they are not enhanced effectively as they were in pNDTI‐TT (Figure S1c–h, Supporting Information). One interesting consideration for the differences between these two systems is the rigid pNDTI‐TT framework compared to pNDI‐TT. pNDTI‐TT is designed to have enhanced backbone coplanarity that makes it more effective at preserving crystallite interconnectivity in aqueous electrolytes. Although the complexity of this is beyond the scope of this current study, it is possible that the crystallization retention supports the counterions adopting the preferential edge‐on location in pNDTI‐TT. Backbone structure and glycolated sidechains—that provide space for the counterions to get too close to the conjugated backbone—may be further explored as optimizable variables, for producing successful, effective doped‐OECT and doped‐OMIEC systems.

## 3 Conclusions

Overall, a new chemical n‐dopant and design rule are identified for n‐type OECTs and OMIECs. We find that TBA‐OH enhances transconductance, mobility, and volumetric capacitance in pNDTI‐TT OECTs, that are representative of key performance metrics underpinning a broad variety of OMIEC technologies/applications.^[^ ^13^ ^]^ This is important because OMIECs need controllable with diverse performance metrics to move beyond PEDOT:PSS and realize their broad application space. GIWAXS and AFM show TBA‐OH behaves as a morphology directing additive and the UPS and EPR show that TBA‐OH behaves as an n‐dopant, with the OH^−^ acting as an anion transfer n‐dopant.^[^ ^40^ ^]^ While the OECT data suggest a synergistic combination of n‐doping and morphology modifications improves key performance metrics, we also show the dopant counterion (TBA^+^) location impacts polaron delocalization. Namely, if TBA^+^ adopts an “edge‐on” location relative to the conjugated backbone, polarons are less bound, compared to when TBA+ cations adopt a “face‐on” location. This phenomenon is rationalized by increased polaron binding energy arising from the Coulomb interaction when the distance between the counterion and polaron is small, which acts to localize the polaron. We suggest the latter is a new design rule that should be considered as part of the development of doped‐OECT and doped‐OMIEC systems. In addition to introducing a new dopant, this work is the first elucidate design rules for doped‐OMIECs to take a step toward realizing doping as a generic strategy for tailoring performance metrics in OECTs and OMIECs.

## 4 Experimental Section

### Organic Solution Preparation

The pNDI‐TT and pNDTI‐TT polymer solutions were each prepared at 5 mg mL^−1^ concentration in chloroform. The TBA‐OH solution was purchased from Sigma Aldrich in methanol at 1 m concentration. The TBA‐OH was added to the polymer so that the TBA‐OH was present in the polymer solutions at molar percentage concentrations of 20, 40, and 400 mol%. Exactly the same quantity of methanol was present in each polymer solution (including the 0 mol% solution), regardless of dopant concentration, to account for any residual solvent‐blending effects from using chloroform with a small quantity of methanol. Solvent‐blend effects are beyond the scope of this study. Additionally, a high doping concentration (1:1 volume ratio) was used to investigate trends in OECT performance with dopant concentration. After mixing and prior to deposition, the solutions were left overnight in a nitrogen‐filled glovebox.

### Organic Electrochemical Transistor Fabrication

Top‐gate bottom‐contact transistors were fabricated by patterning 10 nm metal Cr adhesion layer followed by 100 nm Au conduction layer on clean Borofloat glass slides, using thermal evaporation, to serve as the source and drain electrodes with channel dimensions 600 µm width and 30 µm length. The electrode patterned substrates were subsequently cleaned in an ultrasonic bath with a Decon‐90 soap and deionized water solution, then acetone, followed by isopropanol alcohol. The top surfaces of the substrates were activated by UV Ozone followed by O2 plasma to maximize the effects of the adhesion promotor (3‐(trimethoxysilyl)propyl methacrylate), prior to depositing a 2 µm layer of Parylene C on the substrates, using an SCS Labcoater 2. A 3 vol% microsoap solution was spin coated onto the Parylene C at 1800 rpm to encourage easy peeling of a second, 4 µm sacrificial layer of Parylene C, which was used to pattern the polymer in the channel. The 600 µm width, 30 µm length channels were patterned by photolithography using SPR 220–7 photoresist, exposed to UV light using a Suss MA6 contact aligner and developed using MF‐26 developer. The channel features for the devices were etched by O2 reactive ion etching (RIE) using a MARCH RIE system. Finally, the polymer thin films were deposited by spin coating 70 µL of prepared doped‐polymer or pristine‐polymer solution on clean UV ozone‐activated OECT substrates at 500 rpm for 15 s, followed by 700 rpm for 30 s. Finally, the sacrificial top layer of Parylene C was peeled off to pattern the polymer within the OECT channels.

### Organic Electrochemical Transistor Electrical Measurement and Analysis

The OECT current–voltage characteristics were measured in ambient conditions using a KEYSIGHT B2912A Precision Source/Measure Unit. A 0.1 m aqueous NaCl solution was used as the electrolyte for the OECTs, with an Ag/AgCl gate electrode. The operational stability tests were carried out by fixing *V*_G_ and *V*_D_ at 0.4 V, and applying both for 2650 s. The comparative output and transfer characteristics were normalized for their respective film thicknesses to account for the effects of channel thickness on the OECT current.^[^ ^27^ ^]^ The threshold voltage was extracted from the relationship between √*I*_D_ and gate voltage (i.e., √*I*_D_ and *V*_G_), as measured from the transfer curves.

### Electrochemical Impedance Spectroscopy (EIS) and Capacitance Voltage

EIS was used to determine the thin‐film capacitance values. Impedance spectra were taken from 50 µm × 50 µm Au electrodes coated with the n‐doped and pristine polymer thin films, which functioned as the working electrodes in 0.1 m NaCl aqueous electrolyte solution. An Autolab potentiostat was used to take the measurements in ambient conditions, using a Ag/AgCl pellet as the reference electrode and a Pt wire as the counter electrode, with a 10 mV sine wave at frequencies from 1 × 10^5^ to 0.1 Hz and a −0.5 V DC offset potential. Cyclic voltammetry data were taken at a rate of 0.05 V s^−1^ for three cycles. The data analysis was then performed using Metrohm Autolab NOVA software. The capacitance was normalized by the film volume to give volumetric capacitance.

### Thickness Measurements

A Dektak Profilometer was used to measure film thickness of the polymer thin films in the OECT channels. The final thickness value was taken as the average from three thickness measurements within the channel.

### Ultraviolet Photoelectron Spectroscopy

UPS measurements were performed using a PHI 5600 UHV system coupled with a hemispherical electron energy analyzer and a multichannel detector with a 5.85 eV pass energy. An Excitech H Lyman‐*α* lamp (E‐lux 121, 10.2 eV emission) was used as the photon source with a 90° mirror (E‐lux EEM Optical Module) and a dry oxygen purge through the beam path between 7 and 10 Torr. A negative 5 V bias was applied to the samples during the measurements.

### Electron Paramagnetic Resonance

EPR spectra were recorded using a Bruker EMX PremiumX at room temperature, with 15 dB microwave attenuation, 100 kHz modulation frequency, and a modulation amplitude of 1.00 G. EPR polymer solution samples were prepared at a concentration of 10 mg mL^−1^ of pNDTI‐TT polymer in chloroform. TBA‐OH in methanol solution was added to the polymer to form a 400 mol% doped pNDTI‐TT:TBA‐OH solution. The same amount of methanol was added to the polymer solution to form the pristine sample. Both samples were left to rest for 24 h before EPR testing. EPR measurements were carried out at room temperature, with identical sample sizes and measurement conditions, to ensure the observed signal increase was only attributed to the addition of the TBA‐OH (Figure S4b, Supporting Information).

### Atomic Force Microscopy

Topographical information and surface roughness measurements were taken using a Cypher S atomic force microscope operating in tapping mode. Thickness measurements were also taken using the AFM and measuring the polymer thin‐film in the channel. Data analysis was carried out using Igor Pro.

### Grazing‐Incidence Wide‐Angle X‐ray Scattering (GIWAXS)

Pristine and 400 mol% TBA‐OH‐doped pNDTI‐TT polymer solutions (5 mg mL^−1^ in chloroform) were spin coated onto silicon wafer substrates at 700 rpm. One set of pristine and doped polymer substrates was soaked in 0.1 m NaCl aqueous electrolyte solution for an extended amount of time (92 h). GIWAXS data were collected for doped and undoped polymer substrates allowed to soak in electrolyte and left in the original state. GIWAXS measurements were carried out at the 11‐BM Complex Materials Scattering (CMS) beamline of the National Synchrotron Light Source II (NSLS‐II), Brookhaven National Laboratory. X‐rays with wavelength 0.0918 nm were shone onto the thin‐film samples at the incident angle of 0.10°. The scattering data were collected by Pilatus 800k detector located 260 mm away from the samples, which were calibrated by silver behenate. The measurements were performed in vacuum with the exposure time of 10 s to minimize the air scattering. The data reduction and analysis were performed by SciAnalysis.

### Density Functional Theory Calculations

DFT calculations were performed for pNDTI‐TT‐ and pNDI‐TT‐based oligomers of five repeat units each, with a long range corrected hybrid functional LC‐*ω*HPBE, optimally tuned with respect to both the HOMO and LUMO, and 6‐31G* basis set. The geometry was optimized for the neutral P and ionized P^−^ oligomers, as well as the oligomer–counterion complex [TBA^+^‐P^−^] to obtain the distribution of the excess charge on the oligomer (polaron delocalization). Charge model 5 (CM5)^[^ ^87^ ^]^ was used to derive the partial atomic charges. The optimized geometries were subsequently employed as the basis for time‐dependent DFT calculations to obtain excitation energies and oscillator strengths, and an artificial broadening of *σ* = 0.3 was applied to each peak to establish the optical absorption spectra. All calculations were performed using the Gaussian 16 Revision A.03 quantum chemical calculation package.^[^ ^88^ ^]^

## Conflict of Interest

The authors declare no conflict of interest.

## Supporting information

Supporting Information

## Data Availability

The data that support the findings of this study are available from the corresponding author upon reasonable request.
