# Supplementary material for: New Chemical Dopant and Counterion Mechanism for Organic Electrochemical Transistors and Organic Mixed Ionic–Electronic Conductors
Source: Adv Sci (Weinh). 2023 Jul 19;10(27):2207694. doi: 10.1002/advs.202207694 (PMC10520668; doi:10.1002/advs.202207694)
Supplement: Supplementary file 1 — Supporting Information [file ADVS-10-2207694-s001.pdf]

ADVANCED SCIENCE
Open Access

Supporting Information

for *Adv. Sci.*, DOI 10.1002/advs.202207694

New Chemical Dopant and Counterion Mechanism for Organic Electrochemical Transistors and Organic Mixed Ionic–Electronic Conductors

*Vianna N. Le, Joel H. Bombile, Gehan S. Rupasinghe, Kyle N. Baustert, Ruipeng Li, Iuliana P. Maria, Maryam Shahi, Paula Alarcon Espejo, Iain McCulloch, Kenneth R. Graham, Chad Risko and Alexandra F. Paterson**

Copyright WILEY-VCH Verlag GmbH & Co. KGaA, 69469 Weinheim, Germany, 2016.

Supporting Information

New chemical dopant and counterion mechanism for organic electrochemical transistors and organic mixed ionic electronic conductors

*Vianna N. Le, Joel H. Bombile, Gehan S. Rupasinghe, Kyle N. Baustert, Ruipeng Li, Iuliana P. Maria, Maryam Shahi, Paula Alarcon Espejo, Iain McCulloch, Kenneth Graham, Chad Risko, Alexandra F. Paterson**

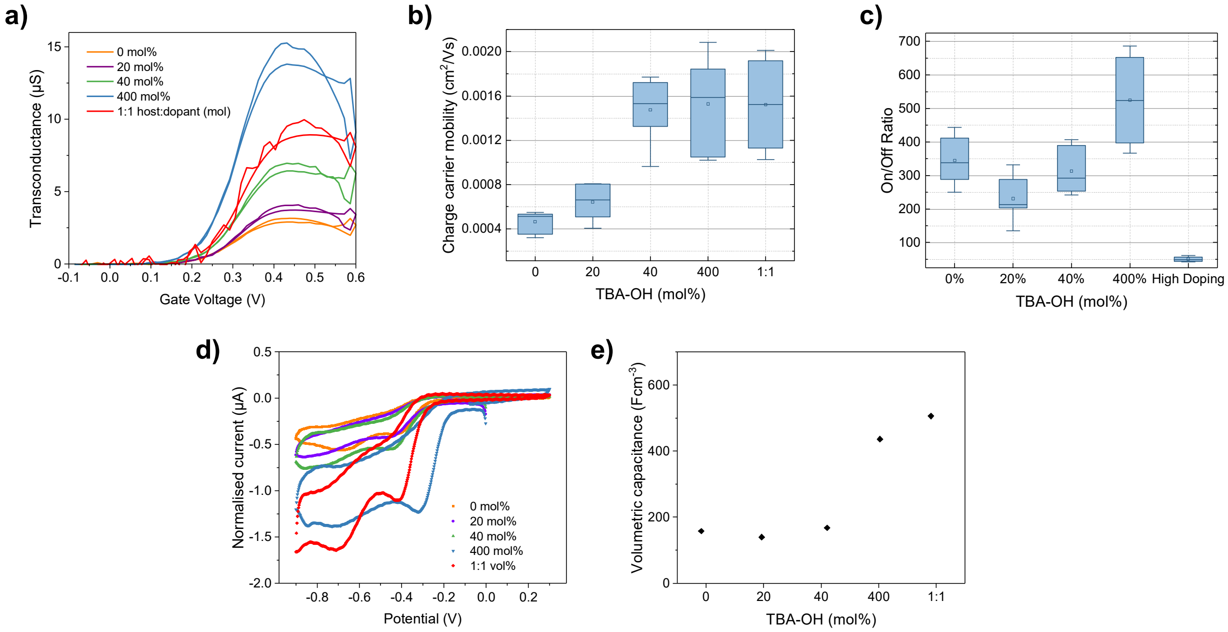

Figure S1. pNDTI-TT OECTs. (a) Peak transconductance plots, (b) Charge carrier mobility, extracted from the peak of the transconductance, in the saturation regime,[9] and (c) ON/OFF current ratio for pNDTI-TT:TBA-OH organic electrochemical transistors, at different doping concentrations (0, 20, 40, 400 mol% and 1:1). (d) Capacitance voltage (CV) measurements using three-electrodes and 0.1 M NaCl aqueous solution and (e) volumetric capacitance, calculated from electrochemical impedance spectroscopy (EIS), showing an increase in C* with TBA-OH doping concentration. Statistics are taken over six transistors for each system. This set is fabricated using one batch of pNDTI-TT, and all devices were made on the same day under exactly the same conditions.

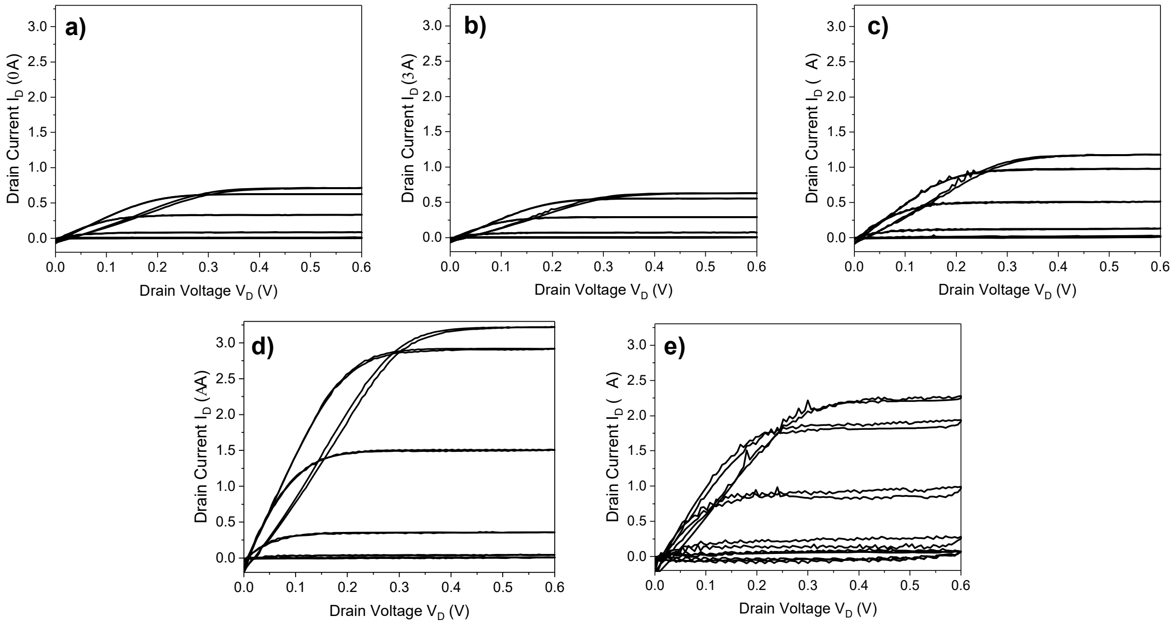

Figure S2. Organic electrochemical transistor output curves for pNDTI-TT transistors containing varying amounts of TBA-OH: (a) 0 mol%, (b), 20 mol%, (c) 40 mol%, (d) 400 mol% and (e) 1:1. The output curves correspond to the transfer curves shown in **Figure 2b**, with channel length 30 μm and width 600 μm. 0.1 M $NaCl_{(aq.)}$ was used for an electrolyte and an Ag/AgCl gate electrode. The active layer thickness values are given in **Table S1**.

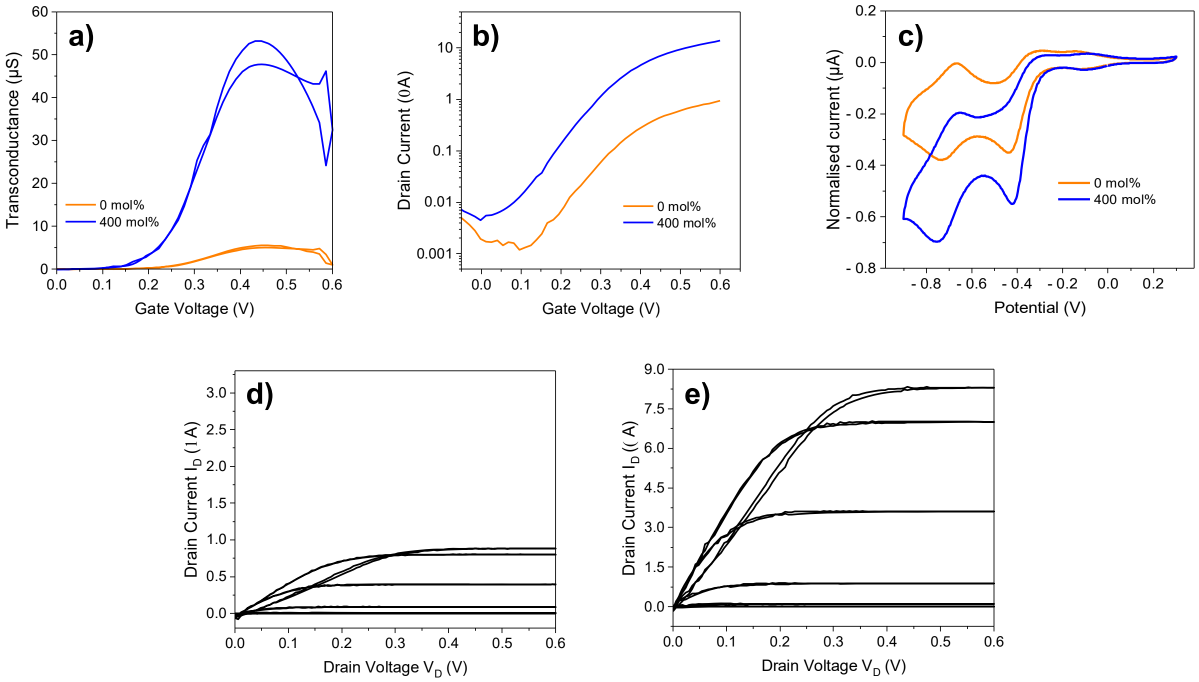

Figure S3. pNDTI-TT and TBA-OH doped pNDTI-TT OECTs. (a) Peak transconductance and (b) backwards sweep transfer characteristics of pristine, 0 mol% (65.4 nm thickness) and the best-performing 400 mol% OECT (47.5 nm thickness) pNDTI-TT:TBA-OH OECTs. (c) Capacitance voltage (CV) measurements using three-electrodes and 0.1 M NaCl aqueous solution. Output curves with V_G: 0V to 0.6 V (d) 0 mol% and (e) 400 mol%. OECT channel dimensions were length 30 μm and width 600 μm. 0.1 M $NaCl_{(aq.)}$ was used for an electrolyte and an Ag/AgCl gate electrode. Statistics outlining device performance are shown in **Table 3**. These devices were fabricated using a second batch of pNDTI-TT, and were made on the same day under exactly the same conditions (i.e. a different batch and day to the devices in Figure S1).

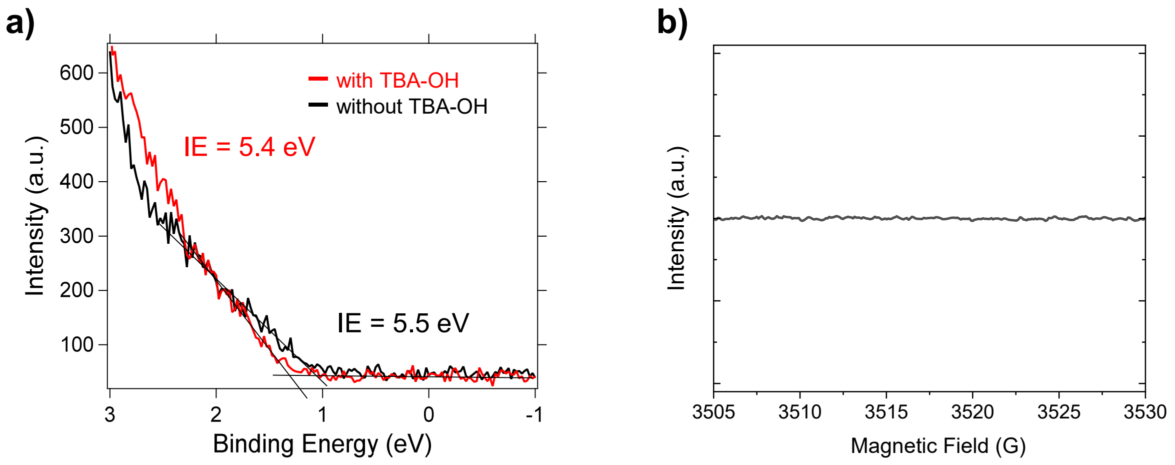

Figure S4. (a) Ultraviolet photoelectron spectra showing the HOMO onset region for pNDTI-TT with and without 400% TBA-OH. (b) Electron paramagnetic resonance (EPR) of TBA-OH only.

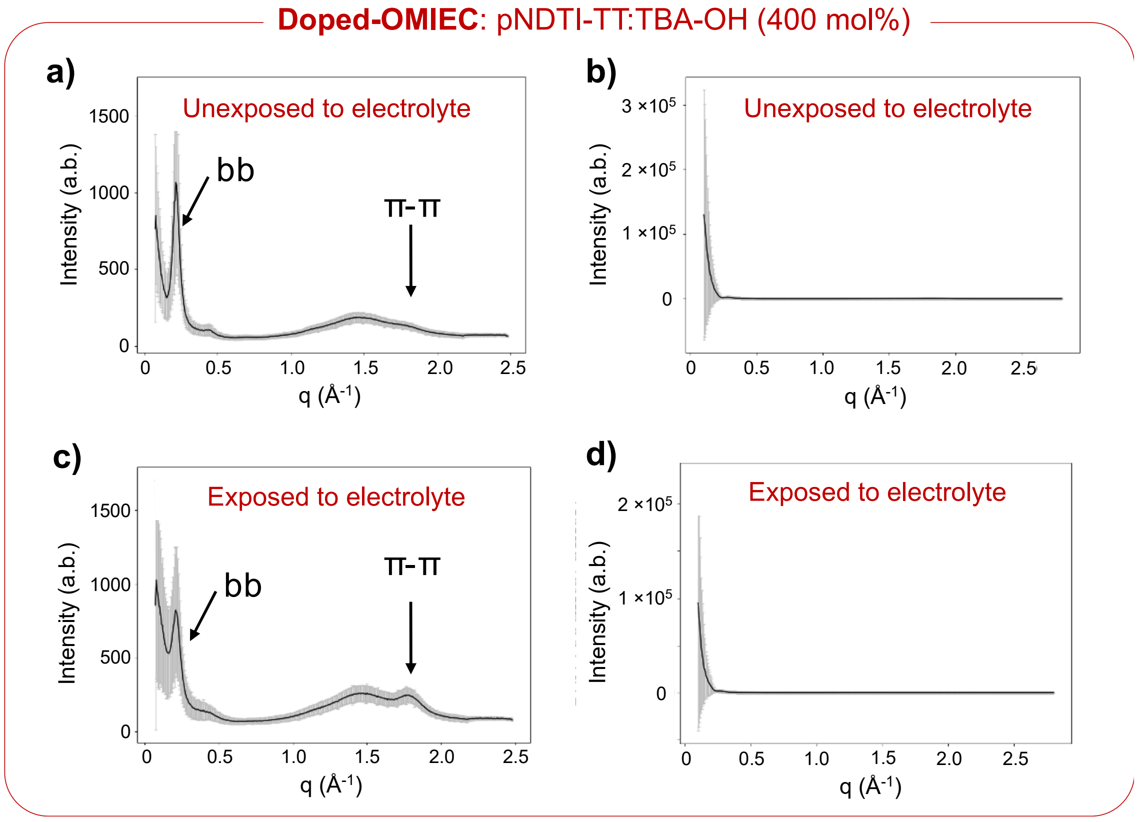

Figure S5. Integrated plots of Grazing-Incidence Wide-Angle X-ray patterns showing (a) horizontal (q_{xy}) and (b) vertical (q_z) directions for best-performing pNDTI-TT:TBA-OH(400 mol%) system that has not been exposed to electrolyte. (c) Horizontal and (d) vertical integration for best-performing pNDTI-TT:TBA-OH(400 mol%) system that has been exposed to 0.1 M

NaCl electrolyte for 3 days prior to measurement. bb and π-π stand for backbone and π-π stacking to guide the scattering peaks, respectively.

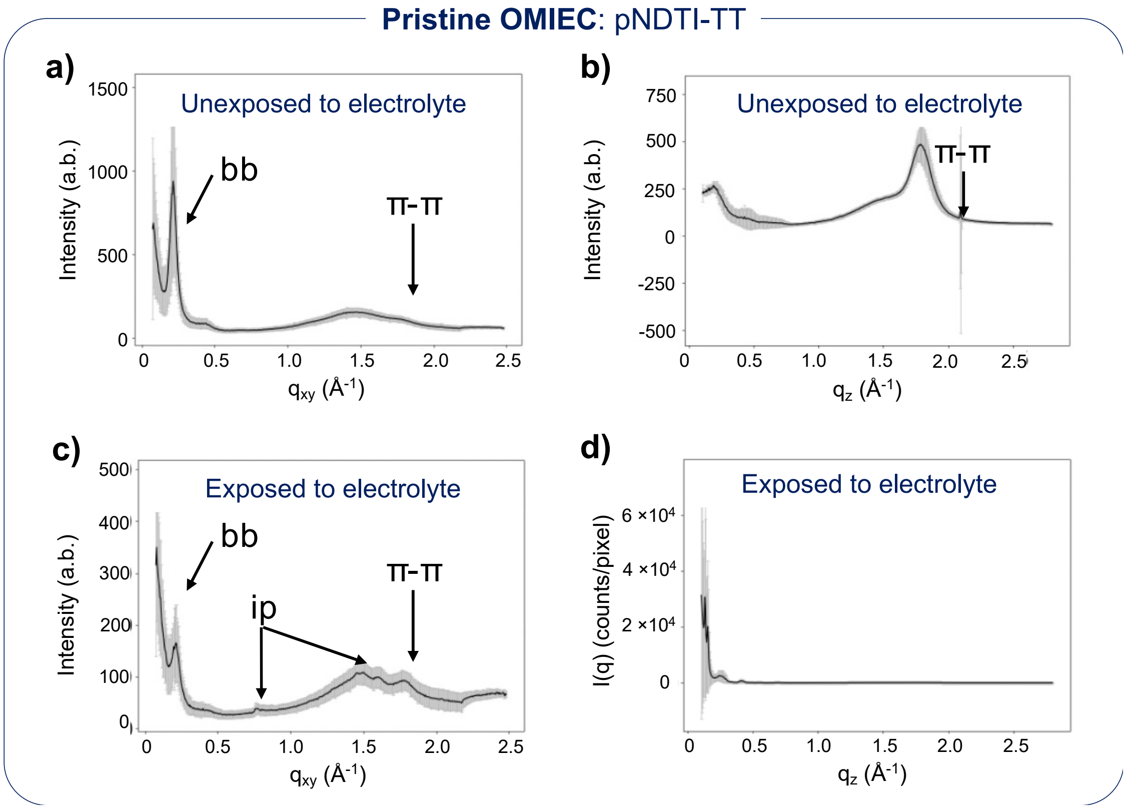

Figure S6. Integrated plots of Grazing-Incidence Wide-Angle X-ray Scattering patterns showing (a) horizontal (q_{xy}) and (b) vertical (q_z) directions for pristine pNDTI-TT without any TBA-OH (0 mol%) that has not been exposed to electrolyte. (c) Horizontal and (d) vertical integration for pristine pNDTI-TT without any TBA-OH (0 mol%) that has been exposed to 0.1 M NaCl electrolyte for 3 days prior to measurement. bb, ip and π-π stand for backbone, the in-plane peak (d=0.82nm and 0.42nm) and π-π stacking to guide the scattering peaks, respectively.

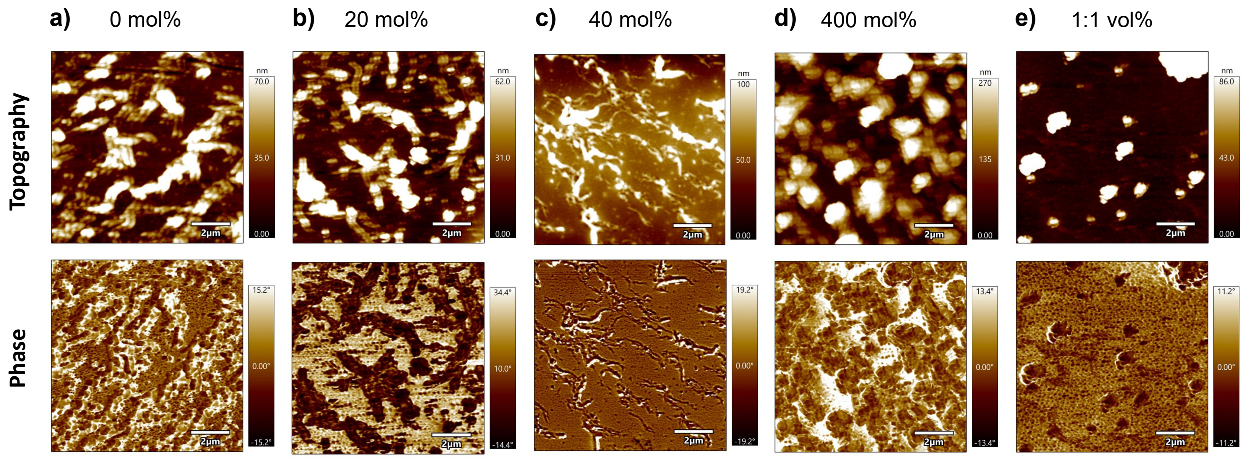

Figure S7. Atomic force microscopy images, showing phase and topography, for the pNDTI-TT thin-films containing varying amounts of TBA-OH: (a) 0 mol%, (b), 20 mol%, (c) 40 mol%, (d) 400 mol% and (e) 1:1. All measurements were taken inside the channel of the best-performing organic electrochemical transistor, per system, and therefore were taken after the system was measured and exposed to electrolyte.

a)

Doping Concentration	pNDTI-TT RMS roughness (10 um scan size)	
	Channel (nm)	Gate (nm)
O mol%	42.062	18.181
20 mol%	41.236	46.525
40 mol%	66.010	92.981
400 mol%	177.570	120.856
1:1 vol%	142.422	109.936

b)

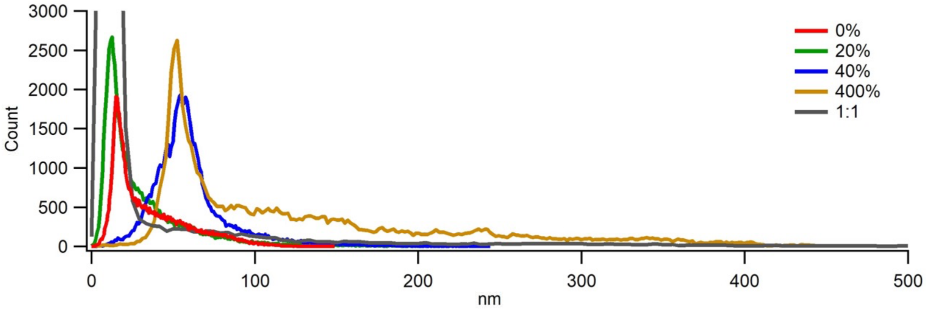

Figure S8. (a) Table of roughness values associated with the atomic force microscopy topography images shown in **Figure S7**, and (b) corresponding histogram.

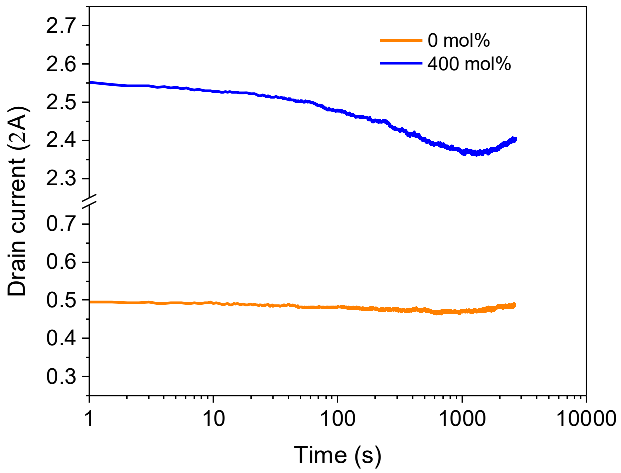

Figure S9. Operational stability of pNDTI-TT and TBA-OH doped pNDTI-TT OECTs. Operational stability was performed by applying constant drain and gate voltages of $V_G = V_D$ = 0.4 V for 2650 seconds.

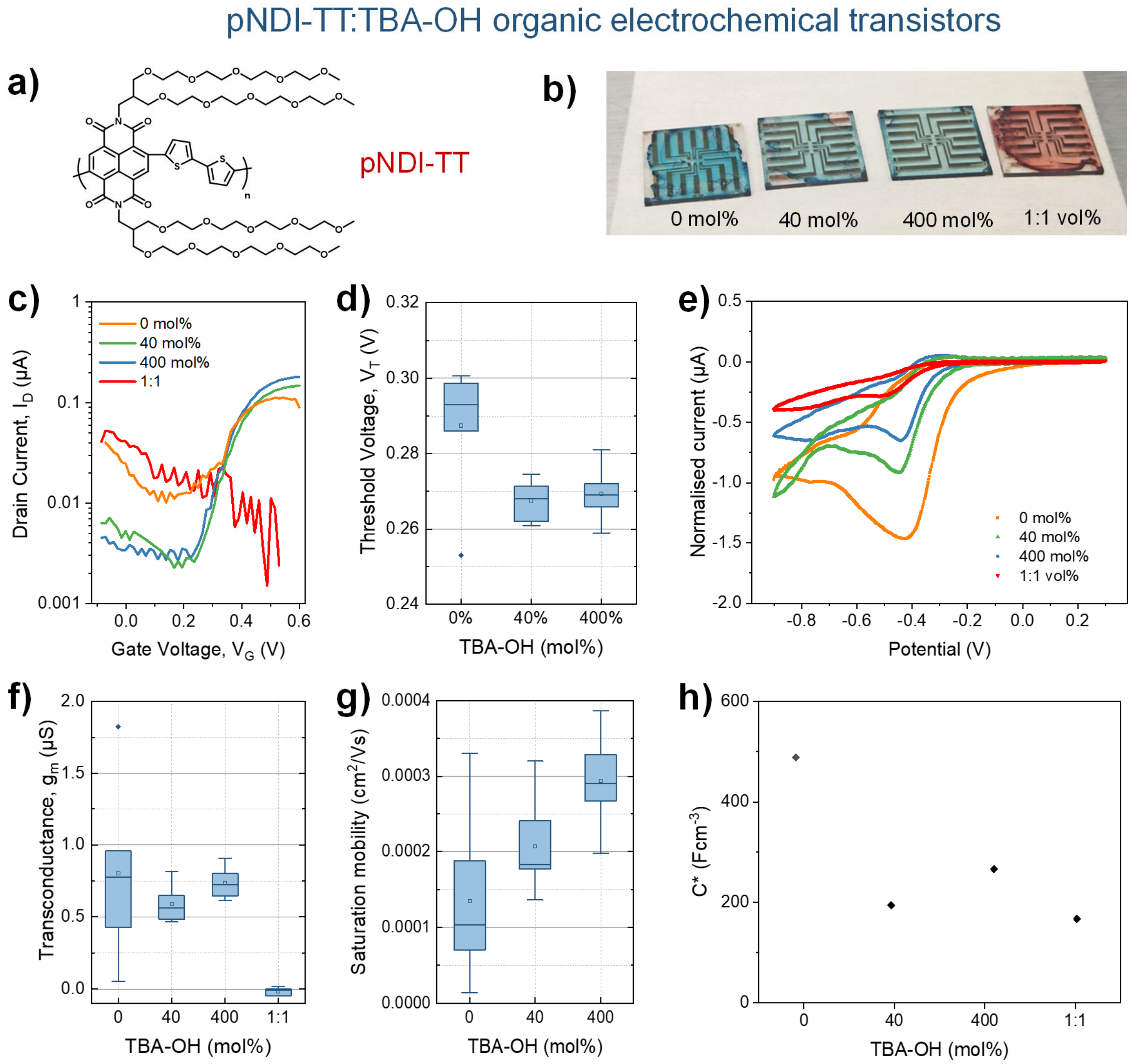

Figure S10. Summary of data for organic electrochemical transistors (OECTs) made with pNDI-TT doped with TBA-OH as the active layer, with TBA-OH at 0, 40, 400 mol% and highly doped 1:1 , to correspond with the pNDTI-TT transistors in **Figure 1**. (a) Chemical structure of pNDI-TT used as the active layer. (b) pNDI-TT thin-film color changing with varying amounts of TBA-OH, indicating adduct formation between the OH^- and pNDI-TT.[48],[45] (c) OECT transfer curves and (d) threshold voltage. (e) Capacitance voltage (CV) normalized for thicknesses given in **Table S1**. (f) Corresponding transconductance and (g) saturation mobility of pNDI-TT OECTs with TBA-OH at 0, 40, 400 mol% and 1:1, where mobility is extracted from the peak of the transconductance.[9] Statistics are taken over six transistors for each system, where highly doped 1:1 isn't included, due to making poor/non-operational devices, as shown in (c). (h) Volumetric capacitance, extracted by fitting electrochemical impedance spectroscopy (EIS) data.

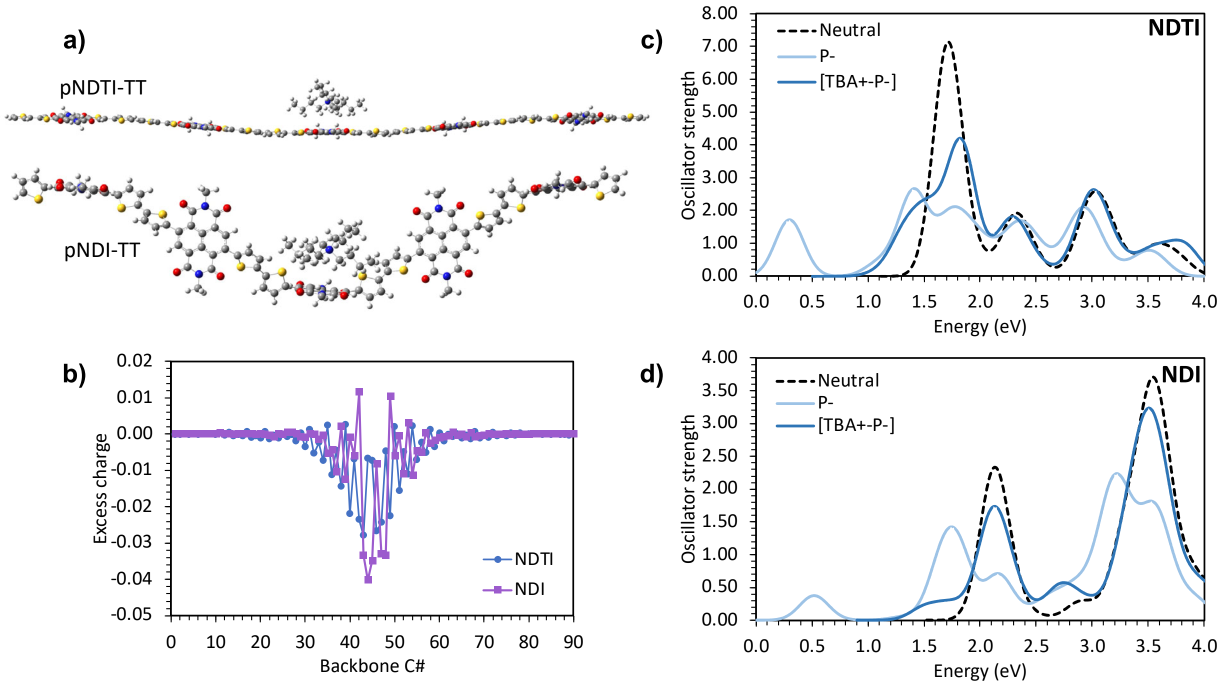

Figure S11. Characteristics of doped pNDTI-TT and pNDI-TT oligomers obtained by DFT calculations: (a) Chain geometry with 'face' location of TBA^+ counterion relative to oligomer; (b) polaron charge distribution (segments of zero charge have been added to both ends of pNDI-TT to match the length of pNDTI-TT); (c) and (d) optical absorption spectrum of reduced chain without and with TBA^+ counterion for pNDTI-TT and pNDI-TT, respectively. The polaron characteristics here are compared for pNDTI-TT and pNDI-TT in their OH^--doped states, with equivalent 'face-on' counterion positions/locations, relative to the chain**. E**xcess charges are found to be more extended and delocalized in pNDTI-TT compared to pNDI-TT, with corresponding full width at half max (FWHM) of 9 and 5 backbone double bonds, respectively. Optical absorption spectra confirms a less bound polaron state for pNDTI-TT, as evidence by the position of the main sub-gap polaron feature. These results are in support of the original study on pNDTI-TT vs. pNDI-TT, that the pNDTI-TT outperforms the pNDI-TT.[36]if both pNDTI-TT and pNDI-TT are n-doped with OH-, then pNDTI-TT system will still outperform pNDI-TT, if both adopt the preferential edge-on counterion location.

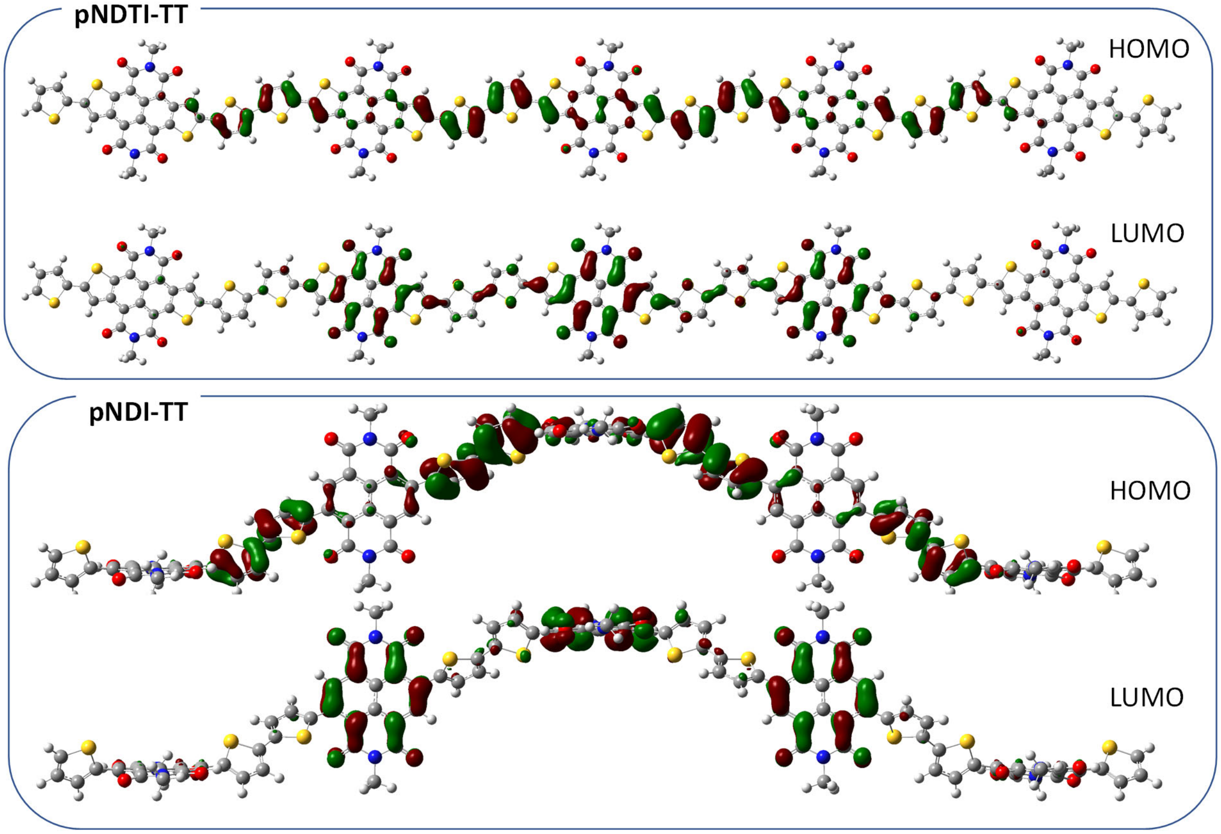

Figure S12. Highest occupied molecular orbital (HOMO) and lowest unoccupied molecular orbital (LUMO) for pNDTI-TT and pNDI-TT oligomers. The LUMO resides mostly on the pNDTI-TT or pNDI-TT acceptor units, while the HOMO resides on the bithiophene donor units.

TBA-OH	Thickness (nm)	
	NDTI	NDI
0 mol%	65.37 ± 2.96	49.65 ± 4.33
20 mol%	63.48 ± 3.48	N/A
40 mol%	43.91 ± 4.61	38.09 ± 4.67
400 mol%	30.08 ± 4.01	24.24 ± 1.92
High (1:1)	20.89 ± 2.12	52.74 ± 12.5

Table S1 Summary of the thicknesses associated with the doped layers, where the thickness is measured over three points from inside the channel (N=3) using a Dektak profilometer. The film-thicknesses are found to decrease with increasing dopant concentration because of the solution doping technique we chose to use in this study, for its simplicity. The thicknesses in this

table are the thicknesses that have been used to normalize experimental data to account for the effects of channel thickness on the OECT current.
